# The potential underlying mechanisms during learning flights

**DOI:** 10.1007/s00359-023-01637-7

**Published:** 2023-05-19

**Authors:** Olivier J. N. Bertrand, Annkathrin Sonntag

**Affiliations:** Neurobiology, Bielefeld University, Universitätstr. 25, 33615 Bielefeld, NRW Germany

**Keywords:** Navigation, Learning, Homing, Hymenopteran

## Abstract

Hymenopterans, such as bees and wasps, have long fascinated researchers with their sinuous movements at novel locations. These movements, such as loops, arcs, or zigzags, serve to help insects learn their surroundings at important locations. They also allow the insects to explore and orient themselves in their environment. After they gained experience with their environment, the insects fly along optimized paths guided by several guidance strategies, such as path integration, local homing, and route-following, forming a navigational toolkit. Whereas the experienced insects combine these strategies efficiently, the naive insects need to learn about their surroundings and tune the navigational toolkit. We will see that the structure of the movements performed during the learning flights leverages the robustness of certain strategies within a given scale to tune other strategies which are more efficient at a larger scale. Thus, an insect can explore its environment incrementally without risking not finding back essential locations.

## Introduction

### Peculiar movements at novel locations

A variety of flying insects, such as bees, often hatch in dark hives that provide them protection from predators and isolate them visually from the outside environment. However, during a certain stage of their life cycle, they need to forage for food sources. In order to feed the colony or offspring, foragers must leave the hive and travel repeatedly between the food source and their home (Klein et al. 2019), due to limited carrying capacities (Leonhardt et al. 2007; Combes et al. 2020). Being naive to the environment outside the hive, they not only need to spend time searching for resources (Reynolds et al. 2007; James et al. 2011), but also make sure they find their way back home. In nature, the colony may reside in the bark of a tree or under the ground [e.g., old mice nest, or in rock crevices—Goulson (2010)]. Even though beekeepers’ hives can be spotted several meters away from the hive entrance, a honeybee needs to locate its own hive and not a neighboring one. Therefore, after a foraging trip, the bee needs to pinpoint where the nest entry is relative to surrounding information to be able to return home. To acquire information about their surroundings, bees and wasps engage in peculiar flights, composed of sinuous movements such as loops, arcs, or zigzags, on their first exits.

When a bee is captured after completing its first flight and released to a location outside the area visited during its first flight, the bee will engage in an extended search. However, if the bee is released within the area visited during its first flight, it will fly straight back home (Degen et al. 2016). Therefore, during these first flights, the bees are learning information guiding them back to their nest, and are, thus, termed "learning flights". These flights are not only performed at the nest, but also at flower sites [e.g., Cartwright and Collett (1983), Lehrer and Collett (1994), Lehrer and Bianco (2000), Robert et al. (2018)], or even by starved non-central place foragers, such as male bumblebees, around flowers (Robert et al. 2017), which may be interested in revisiting the flower later. We will see in this review, how the sinuous movements might support learning at novel locations and how a robust usage of different navigational strategies can be build with experience.

### Orientation and exploration

The sinuous movements at a novel location do not only support learning, but may serve at least two additional purposes. First, these movements are also observed in wasps during the first flight of the day. The wasps spend 50% of its flight time keeping a nearby landmark within 60$^{\circ }$ of its frontal visual field (Zeil 1993). By flying away in a particular direction, wasps may re-learn what they may have forgotten overnight or find a suitable direction along which to fly and seek resources, thus orienting themselves in their environment (Zeil 1993). Similarly, when walking or flying insects are displaced to a novel location, they may engage in a search-like pattern behavior [e.g., Kohler and Wehner (2005)]. This pattern shares similarities with the sinuous movements observed at the nest or flowers, as it is composed of arcs and loops. During this search, it is thought that the animal finds a suitable direction along which to travel, and thus trying to orient itself. We will see that such pattern might also play a role in learning.

Second, over time, the movements at a novel location span larger areas (Osborne et al. 2013; Woodgate et al. 2016; Collett and Zeil 2018). These loops protrude in certain directions of the environment. Therefore, the insects increase the area that has been visited. This increase in coverage allows the insect to explore the environment, on the one hand to learn relevant information, on the other hand to seek suitable resources (Woodgate et al. 2016). Thus, the sinuous movements at novel locations that extend at a large scale, support learning, orientation, and exploration.

### Inside a cockpit of an experienced forager

**Fig. 1:**
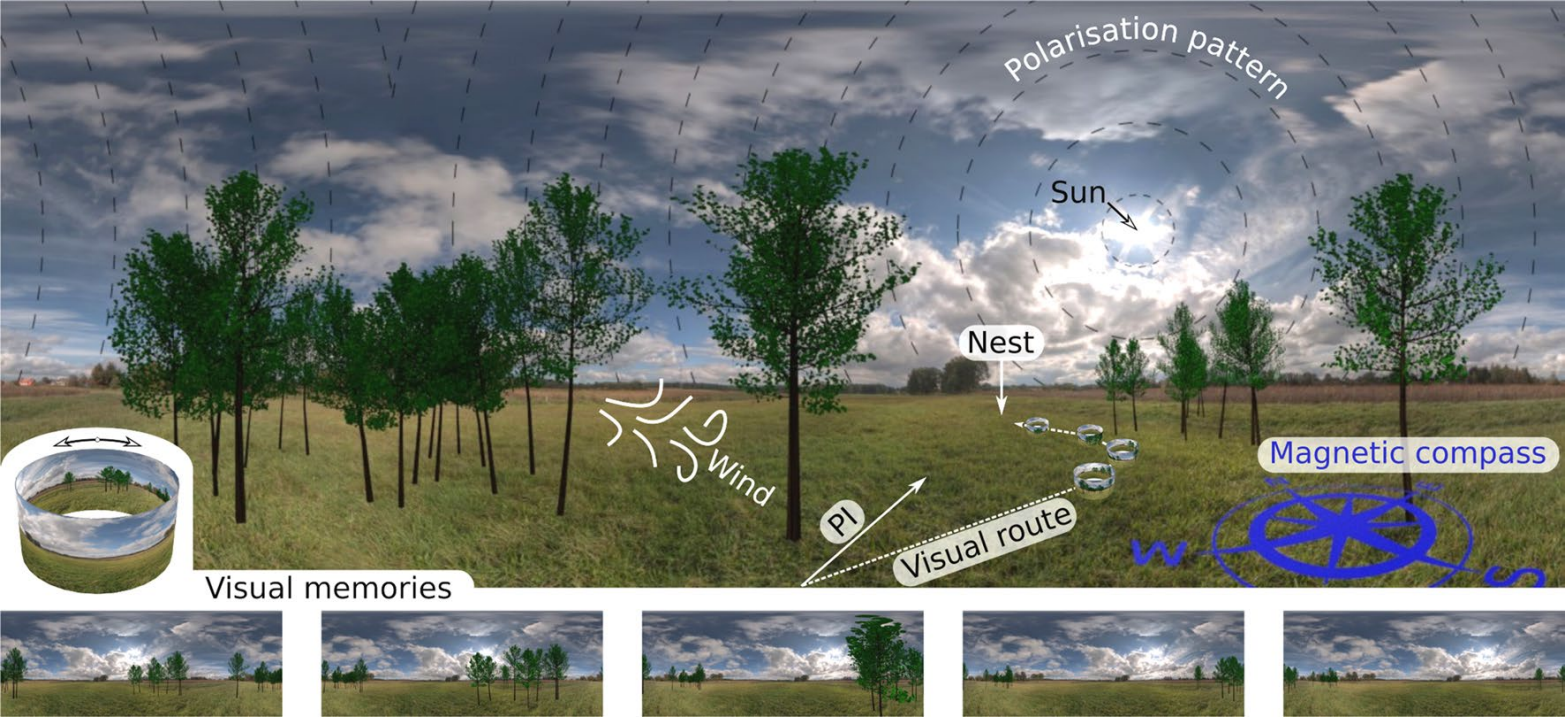
Cockpit view of a multisensory navigation toolkit. Directional cues, such as the sun’s location, the pattern of polarized light, the magnetic compass, or the wind, provide a sense of global orientation for insects. On their outbound journey, path integration (PI) keeps track of their starting location and provides a straight line to that location. A multisensory scenery, represented here by visual information, might be stored at multiple locations along the journey and around the nest, enabling insects to return home

In experienced forgers, the sinuous movements are rarely observed in walking [e.g., Kohler and Wehner (2005), Wystrach et al. (2020)] or flying [e.g., Woodgate et al. (2016), Osborne et al. (2013)] insects. These foragers travel directly between two locations (Woodgate et al. 2016) or, in the case of honeybees and bumblebees, between multiple locations (Lihoreau et al. 2010; Buatois and Lihoreau 2016). In recent decades, these direct paths have been explained by parsimonious and multisensory models that formalize the potential navigational toolkit of insects [e.g., Sun et al. (2020)]. Experienced foragers are thought to efficiently leverage this navigational toolkit by combining several guidance streams and sources of information to achieve robust navigation in various environments. Before diving into the potential combinations, their advantages and limitations, and how the movements described above allow foragers to establish such combinations, we must first examine the cockpit of an experienced forager (Fig. 1). The efficient use of this cockpit is the ultimate goal of a naive insect.

An experienced forager optimizes its journey between locations and may follow an idiosyncratic route [e.g., Kohler and Wehner (2005)]. This route may be guided by visual or olfactory cues that are stable enough in the environment to form a landscape of potential cues [e.g., Sommer et al. (2022)]. Visual information, such as achromatic or chromatic information within the visual field, the skyline (Zeil 2022), or a sense of depth (Dittmar et al. 2010; Doussot et al. 2020; Egelhaaf 2023), learned during previous journeys, allows the insect to follow a previously defined path. This route may be combined with a second guidance strategy: path integration. Path integration results from the integration of distance and direction traveled and yields a direct path to the goal (Heinze et al. 2018). The precision of path integration depends on a precise compass (Vickerstaff and Cheung 2010). Experienced foragers likely possess such a compass by combining different global directional cues coming from magnetic information, the sun’s position, the pattern of polarized light, or the direction of the wind (Heinze et al. 2018). The duo of path integration and route-following will lead the experienced forager close to its goal location, where the pinpointing of the endpoint of the journey will take place. Finally, visual, tactile, olfactory, or vibration information around the goal will guide the insects to this location, i.e., pinpoint the goal (Buehlmann et al. 2020). Throughout its journey, the insect also controls its behavior by avoiding obstacles or threats, regulating its speed, and for flying insects, adjusting their flying altitude. Thus, experienced foragers are efficiently using local control (Ravi et al. 2020), path integration (Webb 2019), local homing (Zeil 2022), and route-following (Bertrand et al. 2021; Buatois and Lihoreau 2016). We will see that these strategies have advantages and limitations (see Sect. 2), and that the structure of the movements observed in naive insects is set to leverage these advantages and limitations (see Sect. 3).

## Interplay between guidances

**Fig. 2:**
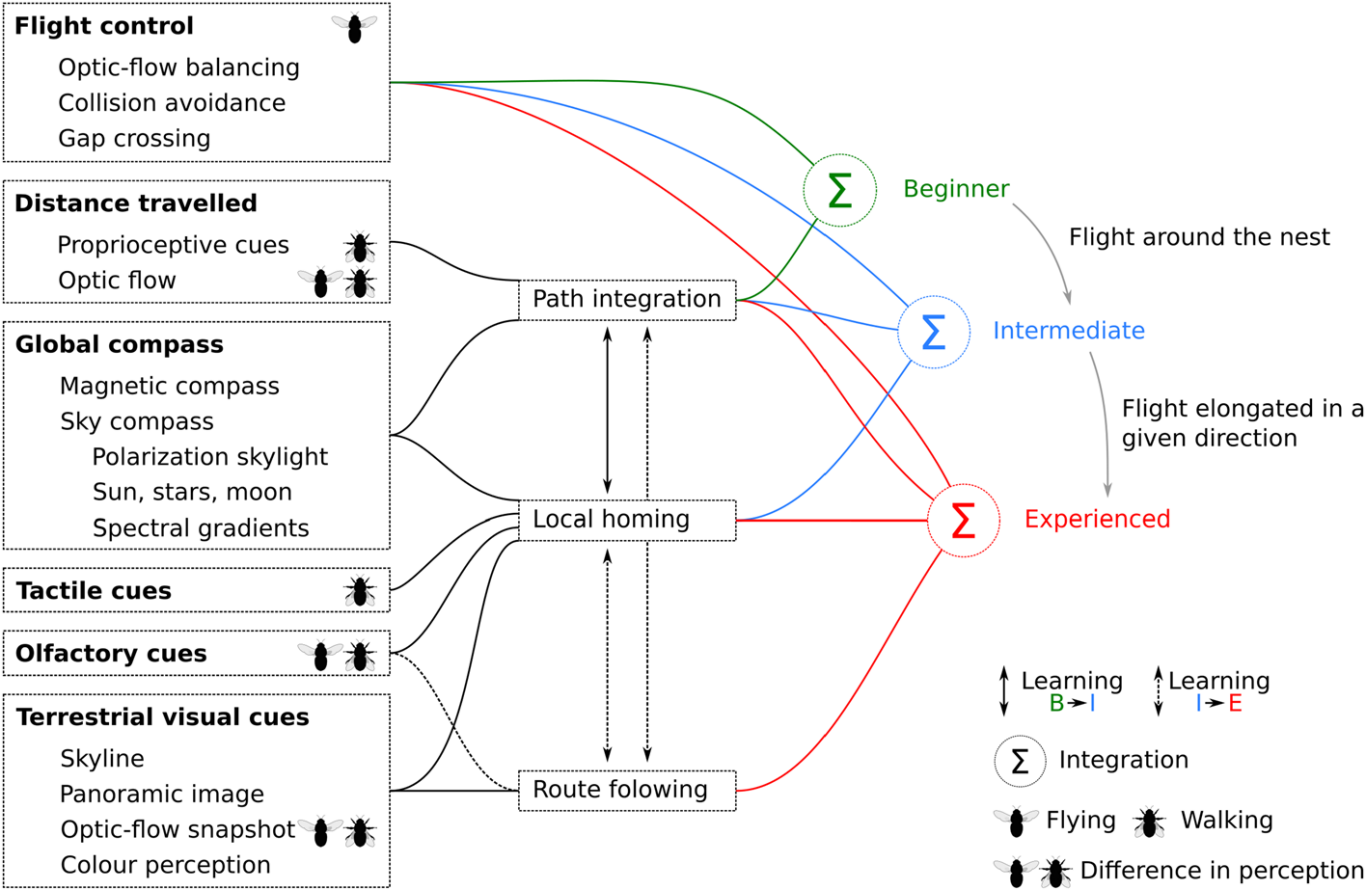
Usage of guidance strategies and their interplays, from naive insects to experienced foragers. The insects’ navigational toolkit can use a variety of sensory information (left) and guidance mechanisms (middle). The interplay between guidance mechanisms varies with experience and scale. One guidance mechanisms might help to tune another one (shown by arrows between guidance mechanisms)

Navigating through the environment can be challenging, especially when the insect needs to integrate different guidance strategies at different spatial scales. In this section, we will explore the interplay between different guidance strategies that enable animals to navigate at both small and large scales. In the next section (see Sect. 3), we will explore how such interplay can be established via experience (Fig. 2).

Insects need to pinpoint their location accurately to find their way home, while at a larger scale, they need to navigate between different locations, such as between their nest and a flower patch, or multiple flowers. Local-scale navigation and global-scale navigation have different requirements and constraints. Pinpointing a location requires a mechanism that allows the animal to converge from surrounding locations around the goal location. This omnidirectional convergence can notably be achieved by path integration but also by using the visual scenery at the goal location and/or nearby locations (Heinze et al. 2018; Zeil 2022). Without omnidirectional convergence, the animal may be able to converge from one side [e.g., by following an odor plume—Steele et al. (2023)], but if it overshoots the goal, the guiding mechanism will lead the insect astray.

At a larger scale, navigation requires a mechanism that allows the animal to follow an efficient path, whether it’s a straight line between two locations in an open field or a tortuous path meandering between objects in the environment. Path integration supports navigation along a straight line toward the starting location from the current location or potentially toward other locations (Webb 2019). Alternatively, the visual scenery surrounding a learned path can guide the animal along this path.

During both homing or following a route, animals still need to react to proximate information to control their trajectories or avoid potential threatening situations, such as avoiding a collision or a predator. In contrast to visual navigation, these local control mechanisms require little learning, and models often use hard-coded mechanisms to mimic such control [Bertrand et al. (2021), Lecoeur et al. (2019), see for reviews Srinivasan et al. (1996), Serres and Ruffier (2017)].

Thus, navigation in insects involves a combination of various mechanisms such as path integration, local control, local homing, and route-following. However, not all of these mechanisms are available to naive insects since some require exploration and learning. To track the transition from a naive insect to an experienced forager, we will start with a duo of mechanisms that require minimal learning, namely path integration and local control. Next, we will explore the duo of path integration and local homing, which is particularly useful for insects that have gained some experience with their environment. Finally, we will incorporate route-following into the integration of various guiding mechanisms, which is beneficial for navigating between known feeding locations and the home. As we progress through these mechanisms, the amount of learning required will increase, reflecting the transition from a naive insect to an experienced forager (Fig. 2).

### Path integration and local control

Path integration and local control are two guidance strategies commonly used by insects for homing (Webb 2019; Heinze et al. 2018). Path integration is a dead-reckoning strategy that provides information about the starting location of the insect, whereas local control helps the insect navigate around obstacles in its environment. The combination of these two strategies can lead to successful homing, but there are limitations to their effectiveness.

The vector from the path integrator only provides information about the direct path back to the starting location. In other words, if the insect encounters obstacles or takes a circuitous route, the vector may point behind obstacles and therefore it cannot provide a direct route back to the starting location. This is where local control comes into play. Local control strategies, such as obstacle avoidance (Bertrand et al. 2015), flight control (Serres and Ruffier 2017), crossing gaps (Baird and Dacke 2016; Ravi et al. 2020?), or wall following, as seen in ants (Collett et al. 2006) and bees (Serres et al. 2008), allow insects to maneuver around obstacles and navigate through complex environments. These strategies rely on the insect’s perception of its immediate surroundings, such as visual cues that inform the insect of the distance and direction of nearby objects (Egelhaaf 2023). By using local control strategies, insects can stay on course, even in the presence of obstacles, and this might be sufficient for an insect new to their environment to navigate (Fig. 2—beginner).

However, the combination of path integration and local control has limitations. For example, local control strategies such as moving away from obstacles until the situation is “safe” enough to use path integration again can lead to the insect getting stuck in a local minimum, preventing it from crossing a boundary to get back to the starting location (Bertrand et al. 2015). In such cases, search-like mechanisms are required to help the insect escape from the local minimum and continue its homing journey. Alternatively, some insects use wall-following strategies to navigate around elongated objects laying on their path. Walking ants, for example, follow the obstacle until path integration can be used again (Collett et al. 2006), allowing them to maneuver around the obstacle while minimizing the required detour. This approach can be effective, but it still requires additional navigation strategies to ensure efficient navigation without detours.

**Fig. 3:**
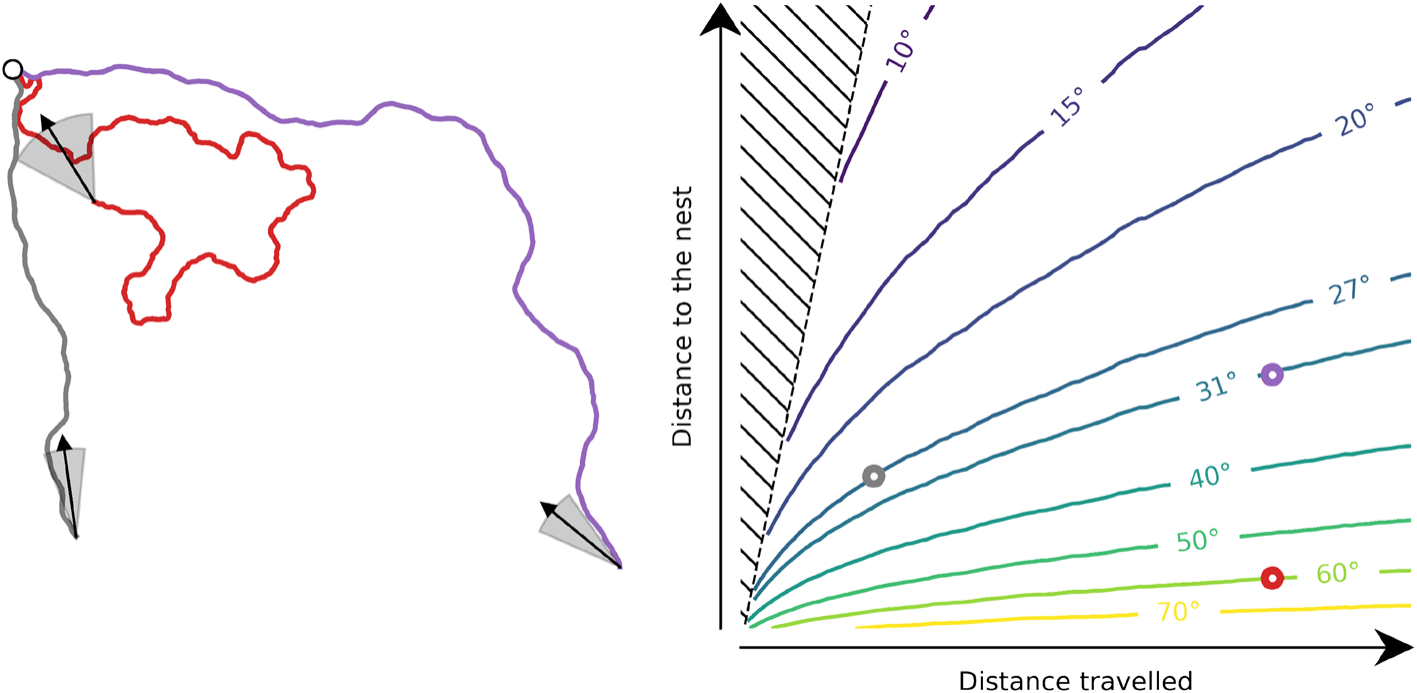
Simulation of directional error given by path integration. The error in the path integrator direction increases with the traveled distance (Vickerstaff and Cheung 2010; Cheung and Vickerstaff 2010). We simulated an agent guided by a directed random walk and derived the error according to Wystrach et al. (2015). Left: three random walks of different lengths are shown. Red and purple are long journeys, whereas the gray is a short journey. Red is close to the starting location. The gray shaded area below the arrow shows the uncertainty of the simulated path integration. The weakly tortuous paths (gray and purple) have been simulated with von Mises with $\kappa =60$, whereas the strongly tortuous path (red) has been simulated with a von Mises with $\kappa =11$. Right: the lines indicate the error in degrees of the simulated path integrator as a function of the distance traveled and distance from the nest. We used 4000 paths for each tortuosity intensity simulated with von Mises distributions of varying concentration (from $\kappa =1$—extremely tortuous, to $\kappa =100$, almost a direct path). We see that the error increases as a function of the distance traveled and decreases with the distance to the nest. The dots indicate the uncertainty of the path integrator of the three example trajectories in the left of the figure

Additionally, when animals travel long distances, the accuracy of path integration decreases as they approach their destination. To illustrate this property of path integration, we simulated an agent following paths of varying tortuosity and length (Fig. 3). Our simulated insect followed a directed random walk composed of unit-length steps interspersed with random rotations following a von Mises distribution. Assuming that the insect is not aware of such rotations and "thinks" it is moving along a straight line, we estimated the directional uncertainty of path integration. As in Wystrach et al. (2015) the uncertainty was calculated as a ratio of distances. The numerator was the deviation from a direct path, i.e., the orthogonal distance to the direct path. The denominator was the traveled distance along a direct path, i.e., without any deviation. Our observations indicate that path integration is less reliable when the insect is close to home after a long journey (Fig. 3, red curve). Therefore, path integration and local control can support efficient navigation at a local scale in two situations. First, away from the goal, the approximate goal direction obtained from the path integrator is sufficiently precise due to the large distance between the current location and the goal (Fig. 3, purple curve). Second, after short travel, the path integrator yield a reliable goal direction, because the traveled distance is short (Fig. 3, gray curve).

### Homing: duo of path integration and local homing

We have seen that path integration provides an animal with knowledge of its starting location and the direct path back to that location, but this guidance mechanism is not precise after a long journey, especially when the animal is in close proximity to its starting location. In contrast, local homing uses information gathered from the environment around the starting location, such as tactile, olfactory, and visual cues (Buehlmann et al. 2020), to guide the insect back to its starting location with a high degree of precision (see cues for local homing in Fig. 2).

Several behavioral experiments have shown the prevalence of visual cues usage in pinpointing a goal location (Tinbergen 1938; Lehrer and Collett 1994; Robert et al. 2018). The return to the goal can be replicated using visual panoramas at the goal location [e.g., walking Mangan (2011), Vardy and Moller (2005), and flying insects Zeil et al. (2003)], or around the nest location [in walking insects Graham et al. (2010), Dewar et al. (2014), Le Möel and Wystrach (2020) and flying Differt and Stürzl (2021)]. These guiding mechanisms, however, only work in a limited range, defined by a catchment area (Zeil et al. 2003). Catchment areas vary between model variants and environmental situations (e.g., with the level of clutter or location of the goal in the environment (Mangan 2011; Müller et al. 2018)). Thus, a return from outside this catchment area requires a different mechanism than homing, which can be accomplished by path integration (Fig. 2—intermediate).

Using a combination of local homing and path integration allows path integration to guide the agent only towards the catchment area. Hoinville and Wehner (2018) modeled this combination by allowing a smooth transition from path integration (distant from the goal) to local homing (nearby the goal). This transition replicated several experiments at various scales (Bregy et al. 2008; Cheeseman et al. 2014; Legge et al. 2014; Wystrach et al. 2015). Although the combination of path integration and local homing can solve the problem of lost precision of path integration towards the home, it does not solve the potential problems encountered away from the goal when obstacles require the insect to follow elongated objects, make detours, or search for an exit from a dead end.

### En route to home: following an optimized path

Away from the goal location, local homing is of little use, and the insect can rely on path integration and local control to reach the vicinity of the goal. However, insects may repeatedly commute between two or more locations, and in doing so, gain the ability to memorize an optimized path that requires little detour or search (Fig. 2—experienced). Behavioral experiments in ants have notably shown visual guidance mechanisms taking place far from the home location. When an ant, deprived of path integration, crosses its habitual route, it will stop searching and follow the route back home (Kohler and Wehner 2005). Therefore, ants are capable of recognizing information experienced along a previously traveled journey away from the nearby environment of their goal. Route-following mechanisms, therefore, complement path integration and local control to navigate efficiently away from home. Parsimonious models using processes similar to local homing can mimic visually guided route behavior [walking: Baddeley et al. (2012), Kodzhabashev and Mangan (2015), flying: Differt and Stürzl (2021)].

Thus, a small set of guidance strategies, including local control, path integration, local homing, and route-following, seems sufficient to allow efficient and optimized navigation between two or more locations at different scales and with different requirements of experience. Experienced foragers can utilize these strategies effectively, but naive individuals require tuning to become experienced. As mentioned earlier, naive and intermediate foragers exhibit sinuous movements that aid in tuning the path integrator (Rössler et al. 2022) and homing mechanisms (Degen et al. 2016). Additionally, as we will see in the following section, these movements could support the tuning of a novel guidance strategy by leveraging the robustness of a mastered strategy.

## Sinuous movements support learning to navigate

We have observed that different combinations of guidance strategies occur at different scales, and also require varying degrees of experience. Naive insects (beginners) might primarily rely on a duo of path integration and local control, which is sufficient for short outbound journeys. Insects with an intermediate experience might leverage information around the goal location, as local homing requires extracting and learning the relevant information at the goal location. The duo of local homing and path integration might be sufficient in various situations, but integrating a route-following strategy allows following optimized paths, particularly in cluttered environments, and extends the homing range of the insect. Insects perform sinuous movements when exploring their environments, such arcs, loops, and zigzags. During these movements, the insects turn back and look at the goal (Lehrer 1991, 1993) or surrounding structure, such as objects (Lobecke et al. 2018), placing the goal or surrounding structure at the highest acuity zone of their visual field (Taylor et al. 2019). We will explore how these movements might support the learning of two duos: path integration and local homing, and path integration and route-following. Furthermore, we will examine the triggers and terminations of these movements.

**Fig. 4:**
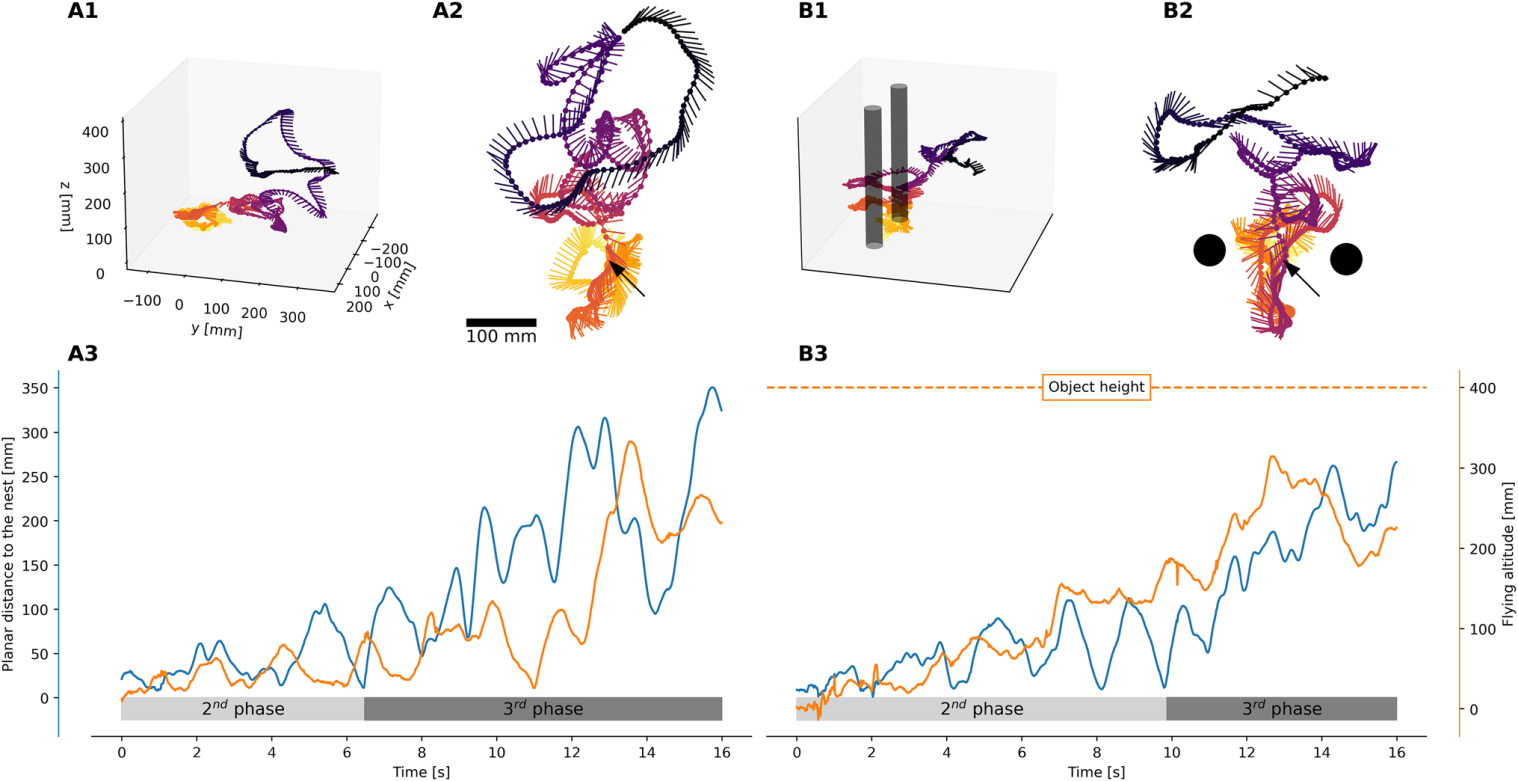
Example of learning flights of bumblebees, *Bombus terrestris*. The first flights of bumblebees was recorded in two environments (Lobecke et al. 2018). The example trajectories are shown in 3D (A1) and in 2D (A2) from a view from above (i.e., looking at the ground) in the first row, where the dots represent the position of the bees’ thorax positions and the lines represent the bees’ orientation. The position of the nest is indicated by the black arrow, and the color indicates the time (yellow for the start and dark purple for the end). The time course of the distance to the nest (shown in blue) and the flying altitude (shown in orange) are shown in the last row. In the flight without objects, the positions of the bees are distributed regularly from exiting the nest to slowly increasing distance and height to the nest (A3). In contrast, the flights where the nest was surrounded by objects (B1 in 3D, B2 in 2D) show an aggregation of positions close to the nest before expanding into wider loops and arcs (B3). The distance to the nest increases over time in all trajectories, but the bees regularly return close to the nest, resulting in the distance getting regularly very small. A similar structure can be seen for the flight altitude, where the bees increase their distance from the floor over time

### Calibrating local homing

Local homing occurs only within the goal vicinity, i.e., within a catchment area. Learning flights around specific locations support the calibration of multisensory local homing, and these flights can be divided into three phases: a walking phase, a flight phase with loops and arcs near the goal, and a flight phase with increasing distance from the nest. The exact structure of these phases depends on the species (Collett and Zeil 2018) and the immediate surroundings. For instance, honeybees fly along arcs when exiting a feeding site from a wall (Lehrer 1991), while bumblebees fly along loops when exiting a nest from the ground (de Ibarra et al. 2009).

During the first phase, insects engage in tiny learning walks on the surface, where they may lay olfactory cues and sense olfactory, tactile, and vibrational cues that may be relevant for later pinpointing the goal. As observed in ants, walking insects learn olfactory, vibrational, and tactile cues in the vicinity of the nest entrance (Buehlmann et al. 2012; Buehlmann et al. 2014). Additionally, experiments on ants show that the loops performed during the walk can help align the sun or polarized compass to the magnetic compass, which is already detectable within the insect nest (Fleischmann et al. 2018). Thus, during this first phase of learning, a flying insect may tune its navigational toolkit similarly to a walking insect, learning proximal cues and adjusting the global compass to ensure the accuracy of the path integrator (Rössler et al. 2022).

In the second phase, bees and wasps fly along loops or arcs in the immediate vicinity of the nest entrance (Fig. 4). Since they lose contact with the floor, tactile, vibrational, and non-volatile cues are no longer sensed. Due to the loops, the insect increases its distance from the nest but repeatedly returns to the nest vicinity. Within this phase, the nest may be visible, at least when the insect is in the vicinity, such as by identifying a black hole on the ground (Samet et al. 2014). This knowledge provides feedback on where the goal is relative to the insect’s position and might be used as training feedback for local homing guidance.

**Fig. 5:**
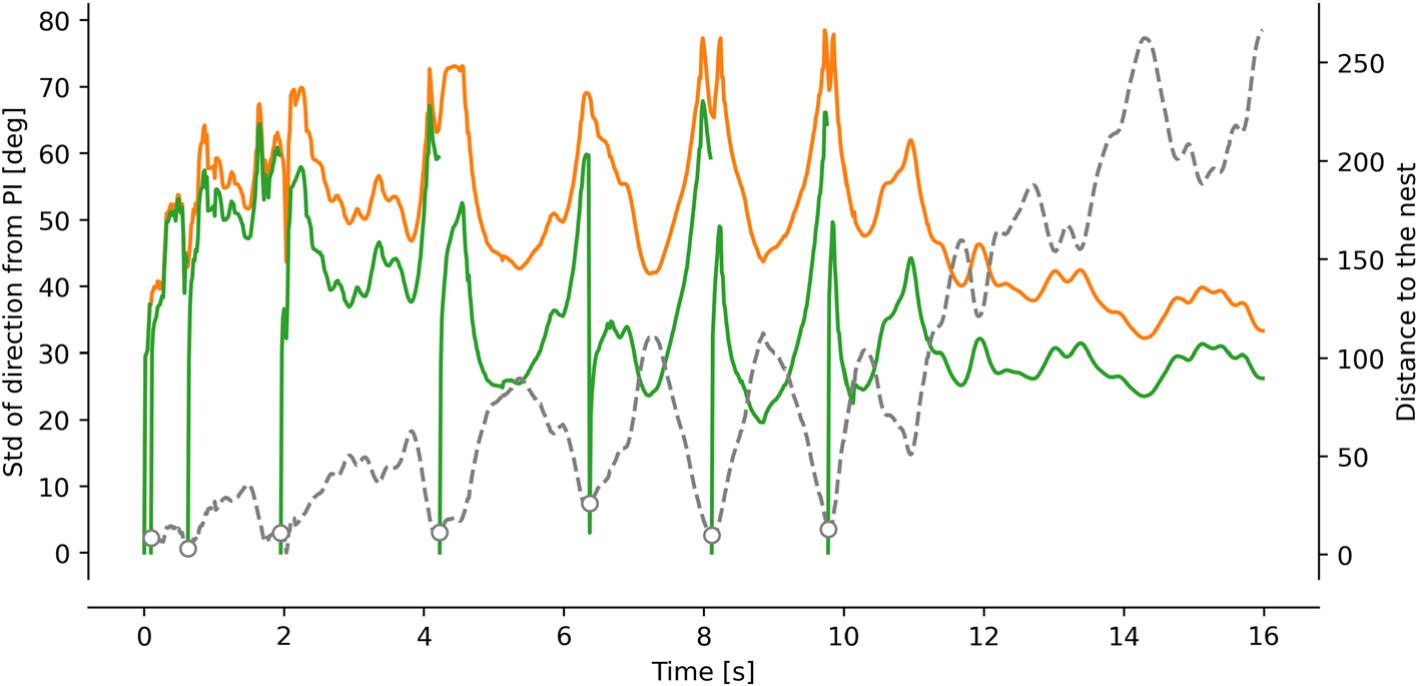
Simulation of directional error along a learning flight. The simulated error of the path integration (as depicted in Fig. 3) along the flight is represented in orange. Additionally, the error of a path integrator that is reset around the vicinity of the nest (indicated by the open gray circle) is shown in green. The distance to the nest is indicated by the dashed gray line

As the third phase begins, the bees continuously increase their distance from the nest, and the arcs and loops become larger in radius, the altitude increases, and the goal may no longer be visible, so it can no longer provide feedback (see Sect. 3.2). However, path integration might be used as an indicator of the entrance position. Indeed, the path integrator always points in the direction of the nest with a certain precision. Due to the tortuous nature of the learning flight, the path integrator might yield imprecise information. Using the simulation in Fig. 3, we estimated this imprecision by using the distance traveled and the distance to the nest during the learning flight (Fig. 5). We observed that the imprecision quickly increases due to the proximity of the nest, but then decreases as the bee gains distance from the nest (orange line in Fig. 5). The imprecision of the learning flight can be reduced by reducing the distance traveled. The return to the vicinity of the nest may be a point in space where the path integrator can be re-initiated, thus resetting the distance traveled and removing its imprecision (green line in Fig. 5).

Therefore, to use path integration as a feedback mechanism to tune local homing, the insect may return to the goal or its vicinity and reset the path integrator. Additionally, the insect may avoid making tortuous loops but move along elongated loops. Thus, at a proximal scale (second phase) path integration and the visual features of the goal can be used as a potential feedback to tune the local homing mechanisms. At a distal scale (third phase), the learning flights structured on elongated loops, allow the use of path integration as a potential feedback to tune these mechanisms at a larger scale beyond the visual proximity of the nest.

### Beyond the local scale: calibrating route-following

**Fig. 6:**
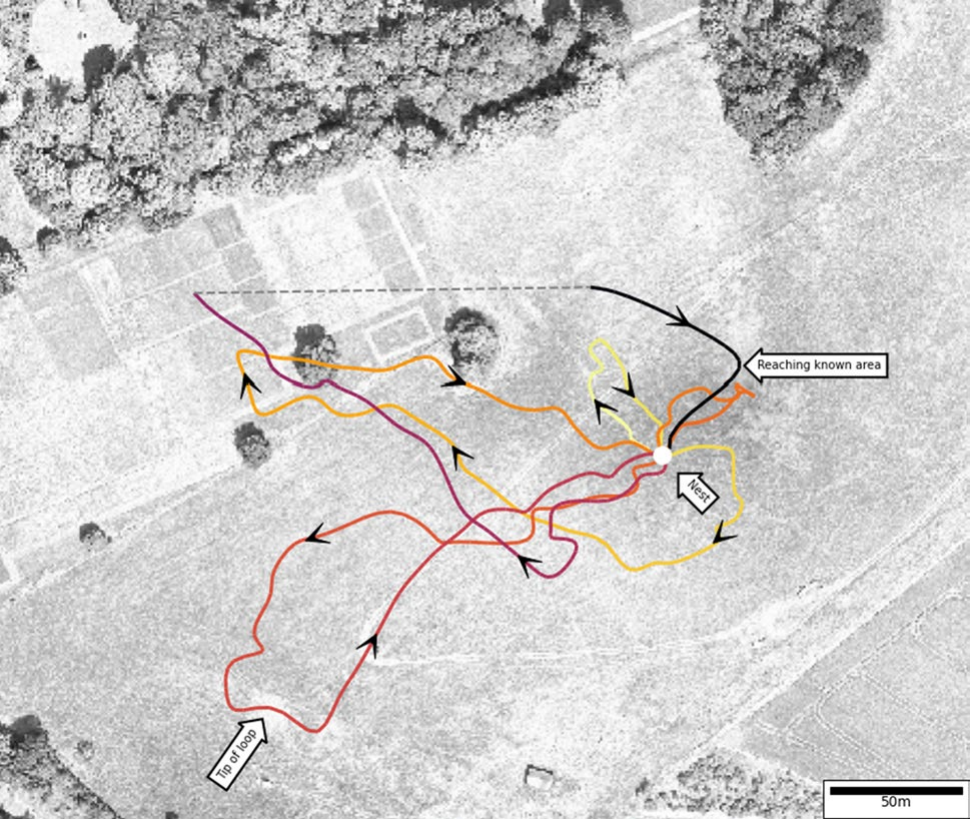
Large-scale learning flight with elongated loops. The nest position is marked with a white dot and the colors indicate the time (light yellow the start and black the end of the flight). The flight contains several elongated loops with straight return to the nest (e.g., red loop with an arrow pointing to the tip of the loop). Arrows indicate the direction traveled of the bumblebee along the flight. Adapted from Woodgate et al. (2016).

The movements of bees during learning flights have been well documented on a small scale, both indoors (Lobecke et al. 2018; Doussot et al. 2021) and in greenhouses (Robert et al. 2018), as well as outdoors (Zeil 1993; de Ibarra et al. 2009). High spatio-temporal resolution cameras are required to estimate the viewing direction of the insect during these flights (Odenthal et al. 2021). Therefore, the movements of bees beyond the local scale have only been coarsely described (Capaldi et al. 2000; Degen et al. 2015, 2016, 2018; Osborne et al. 2013; Woodgate et al. 2016). Nevertheless, at a larger scale, the flights become elongated and protrude in certain directions of the environment (Fig. 6). These protrusions are essential for integrating novel guidance schemes, such as route-following, with known guidance schemes like local homing or path integration.

During elongated flights, the bees travel large distances in a given direction with little deviation from this direction. Therefore, the distance traveled by the bees remains in the same order of magnitude as its distance to the nest. Thereby, the uncertainty of the path integrator remains low. This effect is strongest close to the tip of the elongated loop (Fig. 6). This allows the bee to return to the vicinity of the nest entrance using path integration and local control. When these loops occur at a medium range, path integration is a reliable guidance mechanism. However, the bees will extend the radius of these loops and engage in random searches for flowers over larger distances. After a long loop or random search, path integration is no longer reliable at a medium range. The bees are too far from the nest to use local homing and the distance to the nest is small relative to the distance traveled to rely on path integration. To overcome this problem, bees may learn the visual or olfactory scenery experienced during the elongated flights. Studies have shown that bees use visual information away from the nest to guide their journey at small (Zhang et al. 2012; Bertrand et al. 2021) and large scales (Menzel et al. 2005).

The elongated loops observed in honeybees and bumblebees at a large scale do not necessarily expand in all directions (Degen et al. 2015; Woodgate et al. 2016; Brebner et al. 2021). As a result, a bee arriving from an unvisited location may struggle to return to its home. Thus, the guidance mechanism tuned during these flights is not omnidirectional around the nest. It belongs more to a route-following strategy than a homing strategy [although the underlying mechanism might be fused—Differt and Stürzl (2021)]. Once a bee engages in longer flights, and returns home, these routes can then be followed to guide the bee to the area where local homing is reliable.

The direction of these elongated loop do not appear to be placed arbitrarily in the environment. Indeed, The direction of single elongated loops is shaped by the landscape structures on the ground, in both honeybees (Degen et al. 2015) and bumblebees (Brebner et al. 2021). Following structures on the floor might serve at least two purposes. On the one hand, the structure on the ground might provide a directional cue, in addition to the global compass, and thus reduce the accumulation of error in the path integrator. On the other hand, the ground structure might provide reliable visual features to be used by the visual route-following mechanism.

In conclusion, the elongated loops, shaped by ground structures, provide a solution to maintain a low directional error of the path integration and a chance for the insect to tune its route-following mechanism at a large scale, without getting lost.

### Onset and offset of learning flights

We have seen that the structure of learning flights supports the tuning and calibration of several guidance mechanisms throughout the flights. However, we have not seen when and where such structures must be employed and for how long. At the scale of the entire learning flight, several researchers have examined flight duration. It is clear that new bees, naive to their environment, engage in learning flights. As they leave their hive, the light surrounding them changes from a dark to a bright environment, their mode of locomotion changes from walking in tight spaces to flying, and the untuned navigational toolkit might yield contradictory homing directions, increasing the uncertainty of the integrated homing direction. Additionally, the need to return later may act as a motivation to trigger the performance of such convoluted maneuvers.

Learning flights have been extensively studied at both the nest and flower sites, which are two locations that require later returns. These learning flights may be motivated by the reward associated with a place or the uncertainty within the navigational toolkit. One clear example of the importance of reward in learning flights is observed in male bumblebees. Male bumblebees typically fly directly out of their nest, and do not engage in learning flights at the nest nor flowers. However, when deprived of food and placed on a flower, they will perform learning flights (Robert et al. 2017).

Experienced foragers may encounter difficulties in returning to their goal when there are changes in the environmental surroundings between their departure and return. These difficulties can increase the uncertainty in their guidance strategies, leading to the onset of learning flights later on (Wei et al. 2002; Degen et al. 2018). The uncertainty in guidance strategies may also increase over time between two consecutive learning flights due to memory decay (Menzel and Müller 1996) or abrupt changes in the environment. For example, the turn back and look behavior observed during learning flights at the nest and flower has also been observed in maze-like experiments (Collett et al. 1993) where the scenery changes abruptly due to a wall visually obstructing the previous scenery.

Thus, learning flights are potentially triggered by an increase in uncertainty within the navigational toolkit to navigate, due to internal factors, such as memory decay, or external factors, such as changes in the environments. The movements in the learning flights will stop eventually, questioning the triggers and reasons for terminating a learning flight.

**Fig. 7:**
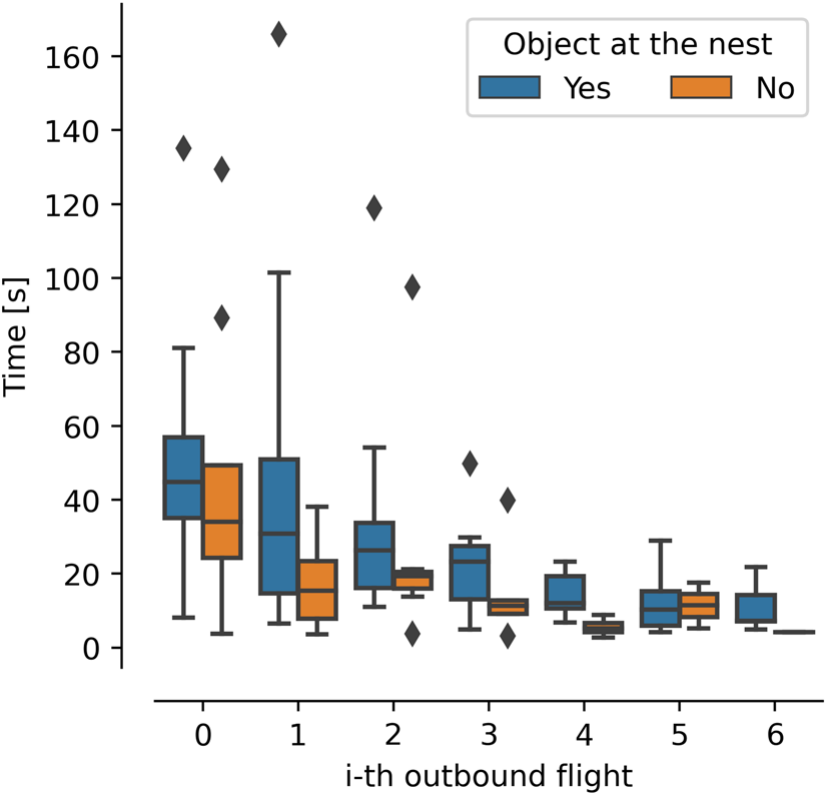
Duration of consecutive learning flights of bumblebees was compared in two environments, as shown in Fig. 4. The nest was not surrounded by objects (in blue) or by two objects (in orange). The data used to generate the figures were obtained from Lobecke (2018) and Bertrand et al. (2020)

By examining the duration of consecutive flights, a significant decay can be observed within a few trials [at a local scale, Wei et al. (2002), Robert et al. (2018), Lobecke (2018); Fig. 7], as well as at a large scale (Woodgate et al. 2016; Degen et al. 2015). During this phase, early working memory is consolidated into long-term memory that the forager will be able to use throughout its lifetime (Menzel and Müller 1996). The duration of single flights varies due to several factors, such as visually challenging environments composed of multiple objects or a temporally unstable landmark, which require longer flights, unlike a simple surrounding consisting of three stable landmarks (Wei et al. 2002). The decay in the flight duration over consecutive learning flights remains unchanged, indicating that the learning rate does not differ between environments (Wei et al. 2002). Moreover, when a bee is not naive to the environment and performs sinuous movements after changes in the nest’s surroundings, the bee covers a larger area than during its first exit (Degen et al. 2018), indicating that re-learning only requires adjusting part of the navigational toolkit. Finally, the duration of the learning flight is also linked to the importance of the location. Bees perform longer learning flights at the nest than at flowers (Robert et al. 2018), and larger bees perform longer flights at flowers with a high sugar concentration than with low sugar concentration while there is no difference in regard to sugar concentration for small bees (Frasnelli et al. 2021). The time bees spend learning has consequences, as the longer they fly, the more accurate they become when pinpointing their goal location (Wei and Dyer 2009). However, longer flights also mean less time available for foraging and finding resources, prompting bees to find a trade-off between foraging speed and accuracy (Chittka et al. 2003), which may explain the differences in learning length observed in simple and complex environments.

As such, the duration and complexity of these flights vary depending on the visual and spatial complexity of the environment, the attractiveness of the location, and the need to balance foraging speed and accuracy. The onset and offset of learning flights are also influenced by environmental changes, memory decay, and the need to return to a location. The onset acts as an indicator of when the insect needs to learn and about the level of uncertainty within its navigational toolkit. The offset indicates the completion of learning, likely resulting in a stable and robust navigational toolkit around a specific location.

## Conclusion

Hymenopterans engage in complex flights that are believed to serve as a platform for learning, orientation, and exploration. These flights consist of unique movements such as arcs and loops, which enable the insect to fine-tune several guiding mechanisms, including the path integrator, local homing, and route-following. The flight structures allow the insect to explore the environment progressively without taking the risk of flying too far and encountering difficulties in pinpointing its goal. Thus, through these flights, the flying insect builds a representation of its environment, enabling it to return to visited goals with great accuracy and over large scales.
